# Supplementary material for: Construction of double reaction zones for long-life quasi-solid aluminum-ion batteries by realizing maximum electron transfer
Source: Nat Commun. 2023 Sep 12;14:5596. doi: 10.1038/s41467-023-41361-z (PMC10497635; doi:10.1038/s41467-023-41361-z)
Supplement: Supplementary file 1 — Supplementary Information [file 41467_2023_41361_MOESM1_ESM.pdf]

Supplementary Information

Construction of "double reaction zones" for long-life quasi-solid aluminum-ion batteries by realizing maximum electron transfer

Zhijing Yu[1,2], Wei Wang[1,2]*, Yong Zhu[2], Wei-Li Song[3], Zheng Huang[1], Zhe Wang[1]*, & Shuqiang Jiao[1]*

[1] State Key Laboratory of Advanced Metallurgy, University of Science and Technology Beijing, Beijing 100083, China.

[2] School of Metallurgical and Ecological Engineering, University of Science and Technology Beijing, Beijing 100083, China.

[3] Institute of Advanced Structural Technology, Beijing Institute of Technology, Beijing 100081, China.

*Email: wwang@ustb.edu.cn (Wei Wang), zhewang@ustb.edu.cn (Zhe Wang), sjiao@ustb.edu.cn (Shuqiang Jiao)

Supplementary Note

Energy Density Calculation

The charge balance of redox reactions in AIBs is different from that of rocking-chair batteries, more similar to that of dual-ion batteries[1-5]. It is known that the total capacity taking into account the mass of the electrolyte in ionic liquid system ($AlCl_3$/[EMIm]Cl) can be calculated based on the following formula[2,6-8]:

$$C_{\mathrm{total}} = \frac{C_{\mathrm{ne}} C_{po}}{C_{ne} + C_{po}} = \frac{Fx(r-1)C_{po}}{Fx(r-1) + C_{po}(rM_{AlCl_3} + M_{[EMIm]Cl})} \tag{1}$$

where F is Faraday constant (26.8 × 10^3 mAh mol^{-1}), x is number of electrons used to reduce 1 mol of $AlCl_3$, r is the $AlCl_3$/[EMIm]Cl molar ratio, C_{ne} and C_{po} are respectively the specific capacity of the actual negative electrode material ($AlCl_3$) and positive electrode material(mAh g^{-1}), and M is the molar mass (g mol^{-1}).

As for the quasi-solid electrolyte system, the molar ratio of $AlCl_3$: [EMIm]Cl: acrylamide is 2:1:0.4. Therefore, the specific capacity of the actual negative electrode material $AlCl_3$ in the gel polymer electrolyte can be expressed as:

$$C_{\mathrm{ne}} = \frac{Fx(r_{AlCl_3} - r_{[EMIm]Cl} - r_{acrylamide})}{r_{AlCl_3} M_{AlCl_3} + r_{[EMIm]Cl} M_{[EMIm]Cl} + r_{acrylamide} M_{acrylamide}} \tag{2}$$

In addition, batteries that use NiTe as a positive electrode material operate based on the following reaction:

$$\mathrm{NiTe{+}3AlCl_3 \leftrightarrow TeCl_3AlCl_4{+}NiCl_2{+}2Al} \tag{3}$$

According to the above reaction, the value of x for NiTe is 2, which means that the NiTe positive electrode requires less electrolyte for the given charge storage. And the total capacity and energy density of a conversion-type Al-NiTe battery are as follows:

C_{total}=69 mAh g^{-1}; Energy density=90 Wh kg^{-1} (average voltage=1.3 V)

For comparison, the energy density of an Al-graphite battery in the same quasi-solid system is also calculated to be 44 Wh kg^{-1} (based on $C_{graphite}$=120 mAh g^{-1}, average voltage=2 V). Meanwhile, it can be concluded from the literature that in the ionic liquid electrolyte system[6], $AlCl_3$/[EMIm]Cl=1.3 is considered the optimal molar ratio for ILs-AIB in most reported papers[9], in which the highest energy density of the Al-graphite battery is 33 Wh kg^{-1}, much lower than that of the Al-NiTe battery with double micro reaction zones after 4000 cycles in this work (80 Wh kg^{-1}). In addition, we can clarify from the literature[7] that the energy density of organic positive electrode material is ~54 Wh kg^{-1}, which is also lower than that of AIBs based on the NiTe conversion-type positive electrode in our work. Therefore, it can be concluded that the quasi-solid conversion-type AIBs are more competitive in energy density due to the less $AlCl_3$ required for the given charge storage and the higher proportion of $AlCl_3$ in the electrolyte. With the construction of DRZs, the LQS-AIBs can continuously and steadily deliver an impressive energy density, demonstrating broad application prospects in the field of large-scale high-safety energy storage.

Supplementary Figures

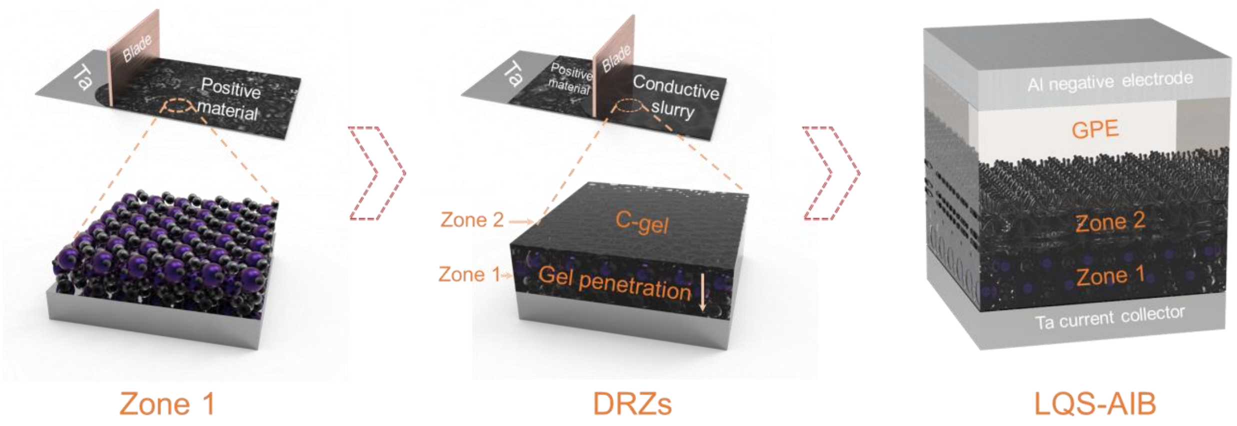

Supplementary Fig. 1. Design strategy of DRZs.

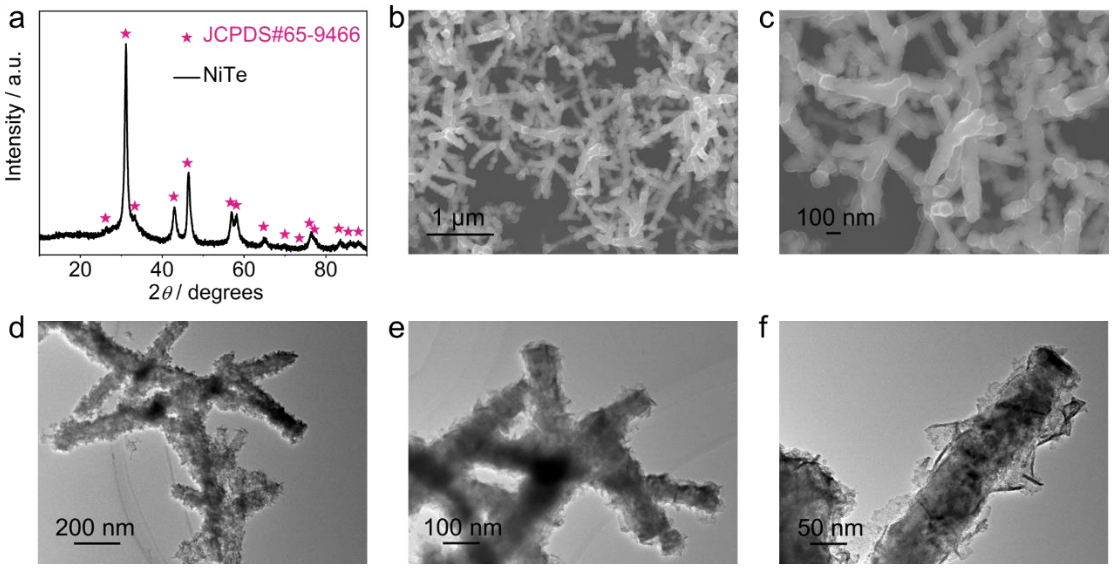

Supplementary Fig. 2. Characterizations of NiTe nanorods. **a** XRD spectra. **b,c** SEM images. **d-f** TEM images.

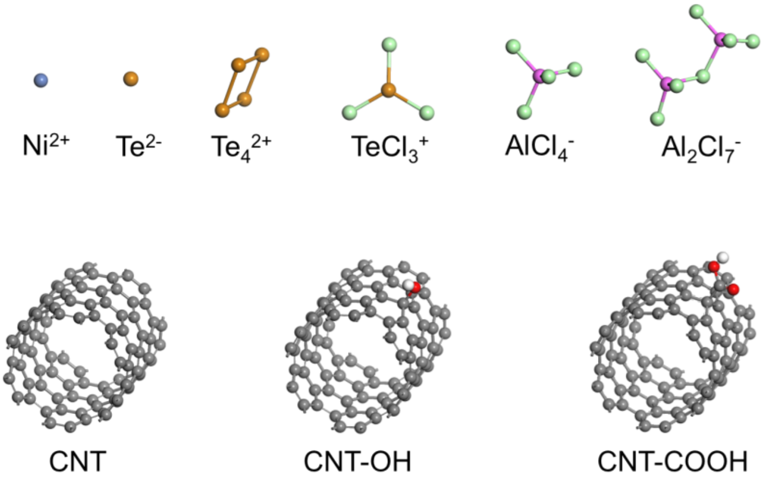

Supplementary Fig. 3. Relaxed structures of the individual models.

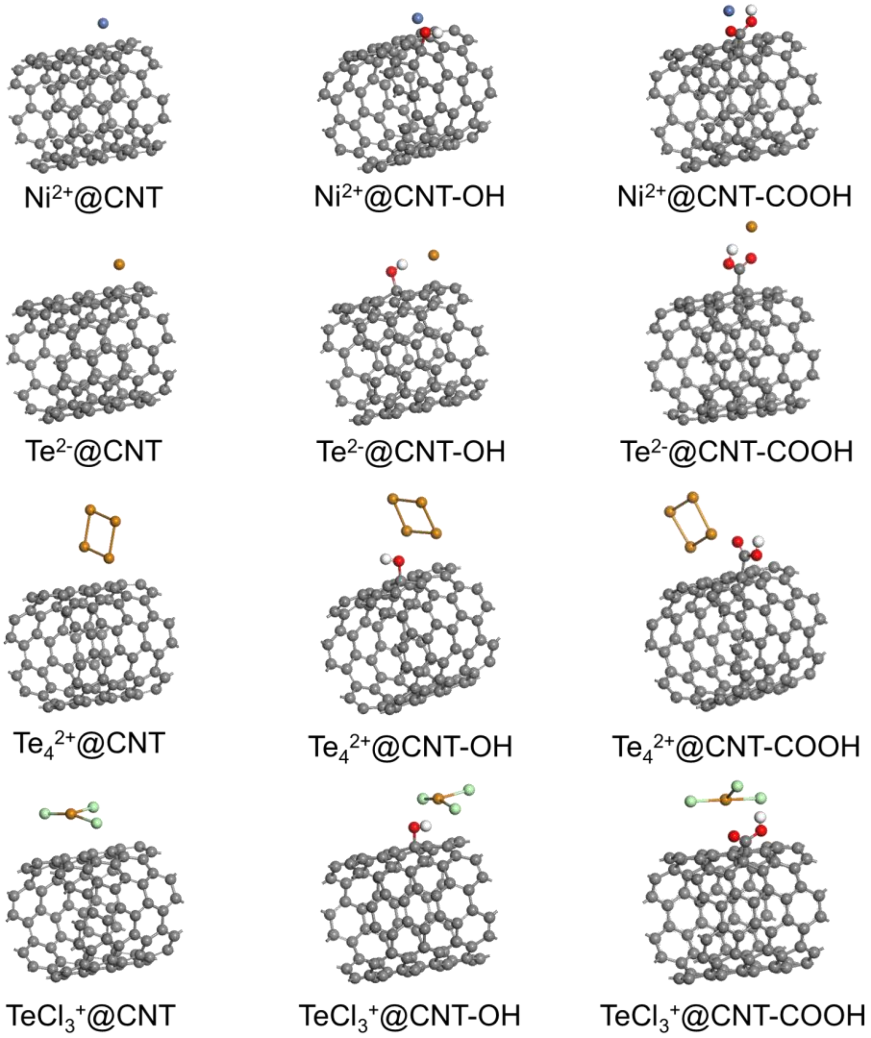

Supplementary Fig. 4. Optimized structures of soluble ions adsorbed on SWCNT (or -OH, -COOH).

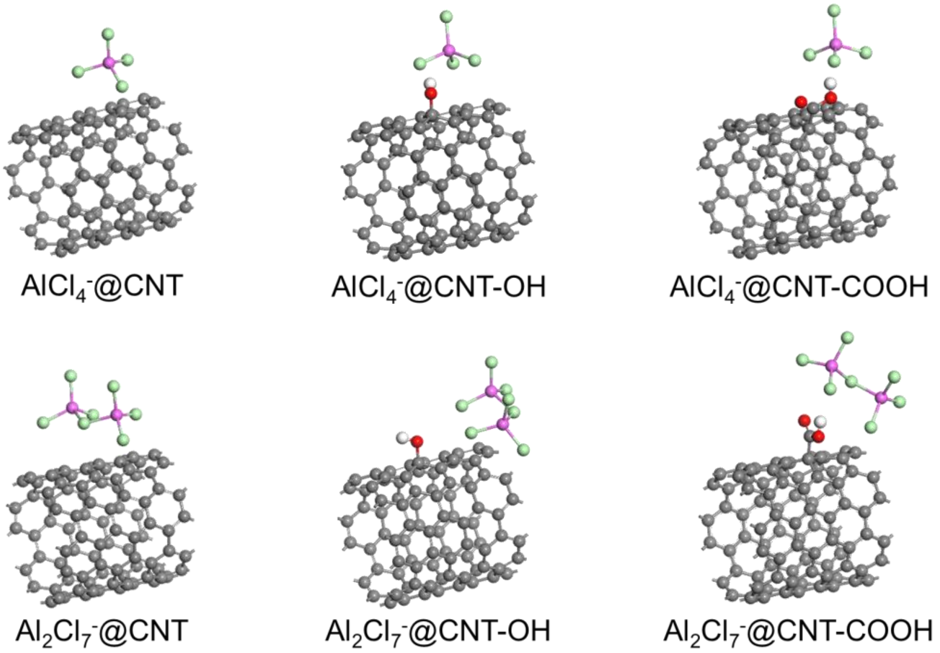

Supplementary Fig. 5. Optimized structures of the major anions in electrolyte adsorbed on SWCNT (or -OH, -COOH).

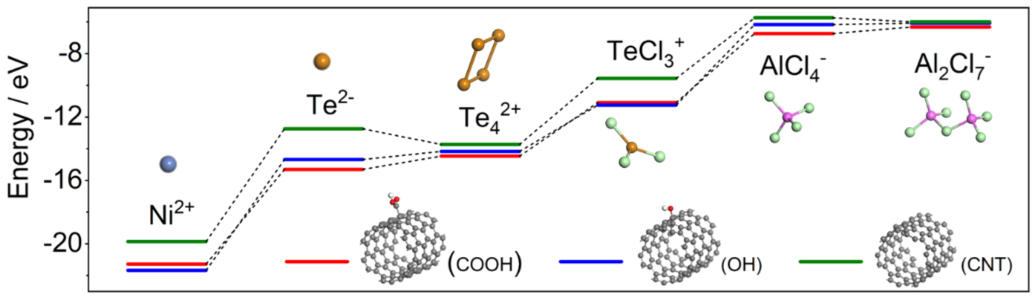

Supplementary Fig. 6. Calculated binding energies of soluble ions in the cell system when adsorbing on SWCNT (or -OH, -COOH).

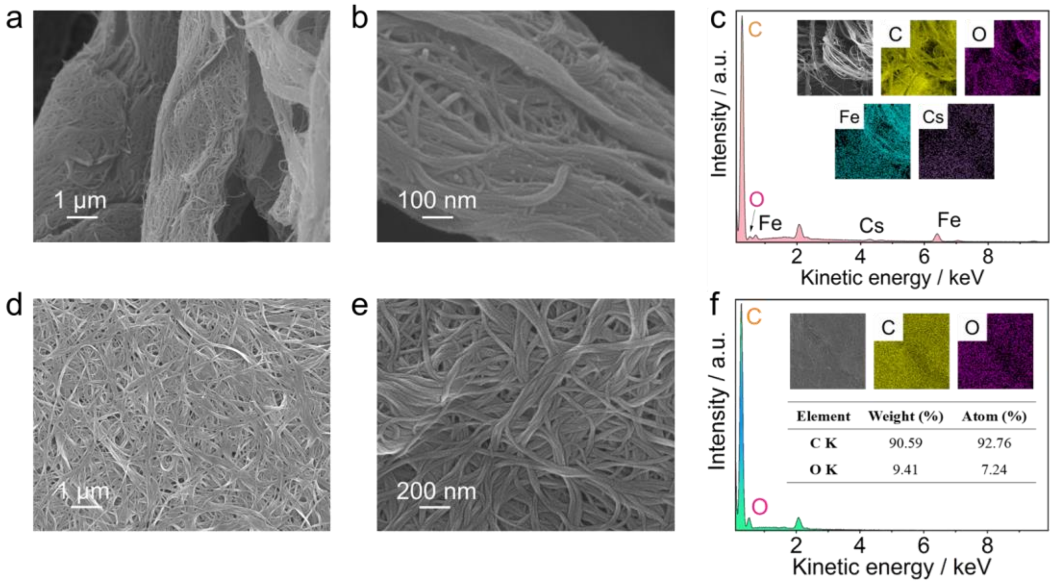

Supplementary Fig. 7. Characterizations of morphology and element distributions of SWCNTs. **a,b** SEM images and **c** EDS spectra of the original SWCNTs. **d,e** SEM images and **f** EDS spectra of the acidified SWCNTs.

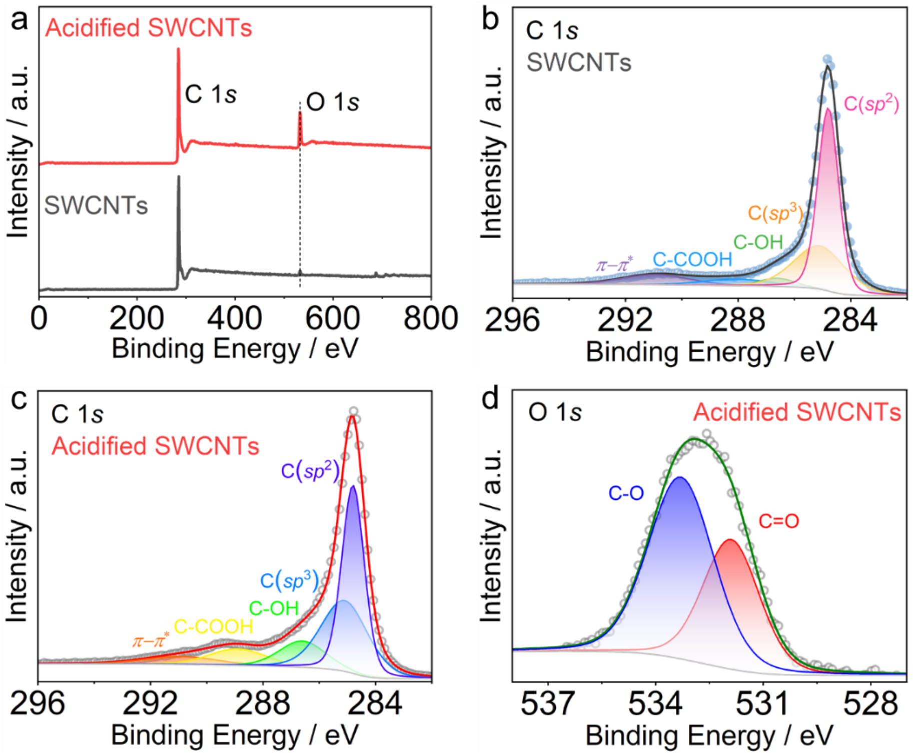

Supplementary Fig. 8. XPS spectra of SWCNTs. **a** Survey XPS spectrum. **b,c** C 1*s* XPS spectra before and after acidification. **d** O 1*s* XPS spectra of the acidified SWCNTs.

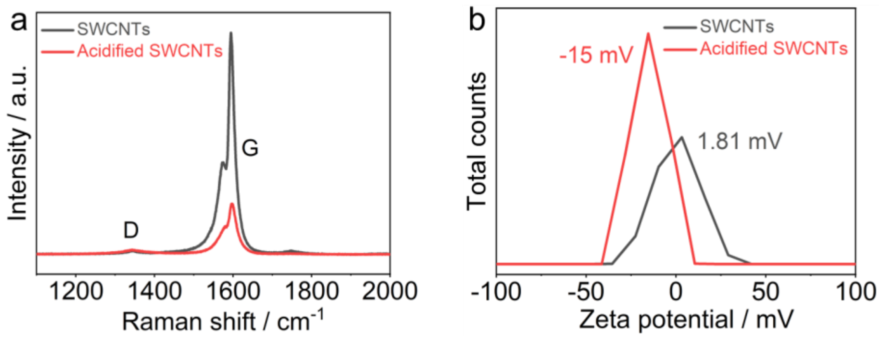

Supplementary Fig. 9. Characterization of physicochemical properties of SWCNTs. **a** Raman spectra and **b** zeta potential of the SWCNTs before and after acidification.

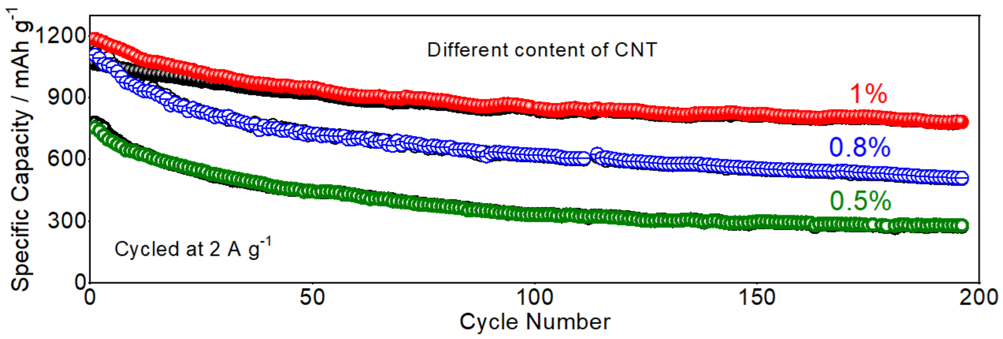

Supplementary Fig. 10. Cycling performance of LQS-AIBs with different content of SWCNTs in Zone 2.

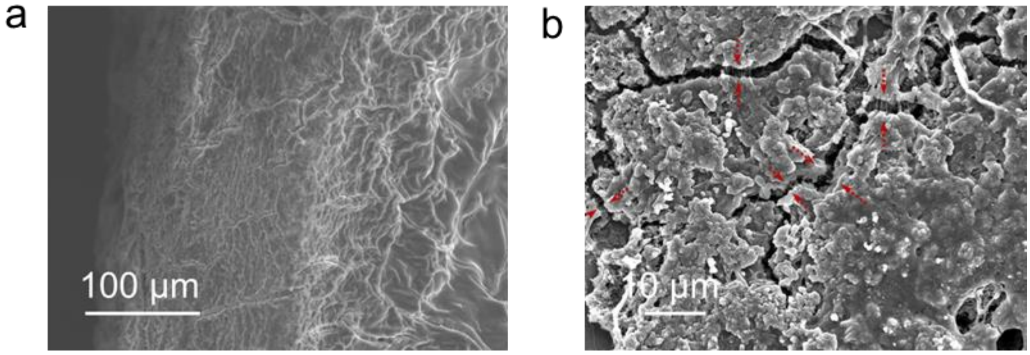

Supplementary Fig. 11. SEM images of Zone 1 after soaking treatment in dichloromethane (DCM).

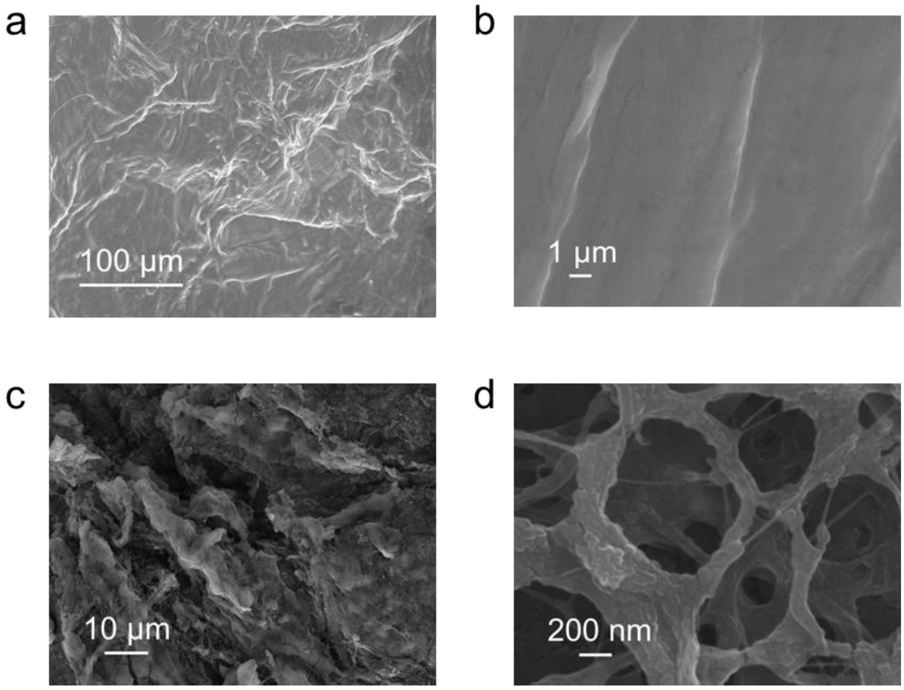

Supplementary Fig. 12. SEM images of Zone 2 **a,b** before and **c,d** after soaking treatment in DCM.

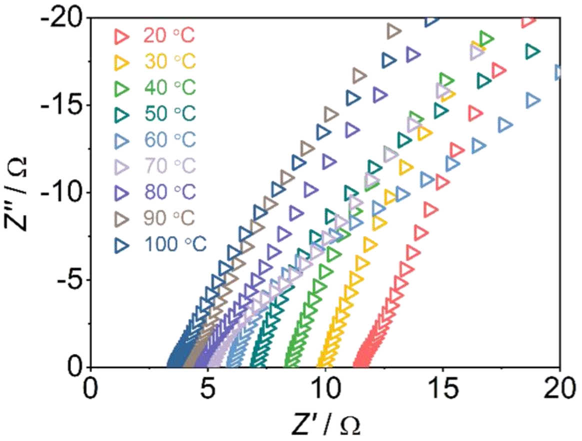

Supplementary Fig. 13. Nyquist impedance plots for C-gel/GPE at temperatures of 25 to 100 °C.

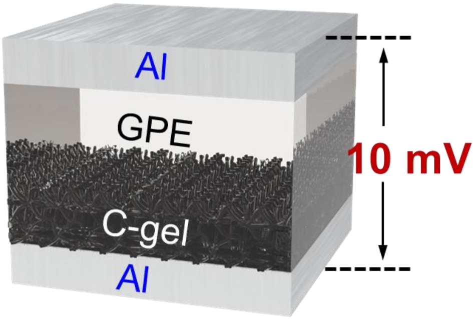

Supplementary Fig. 14. Cell model for the test of transference number (t_-).

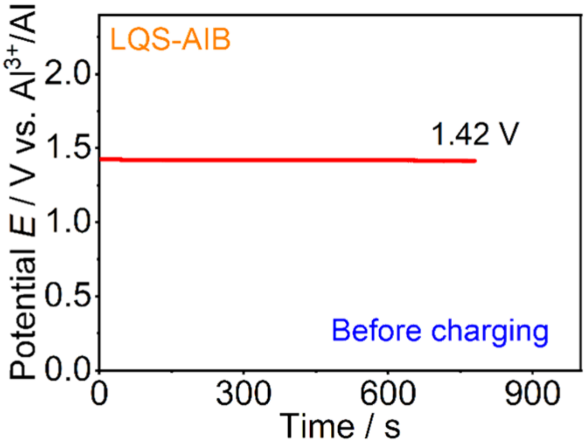

Supplementary Fig. 15. Initial open-circuit potential of an assembled LQS-AIB.

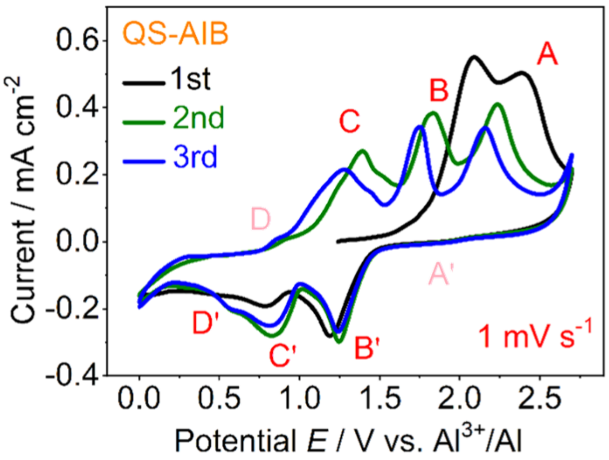

Supplementary Fig. 16. CV curves of QS-AIB at a scan rate of 1 mV s^{-1}.

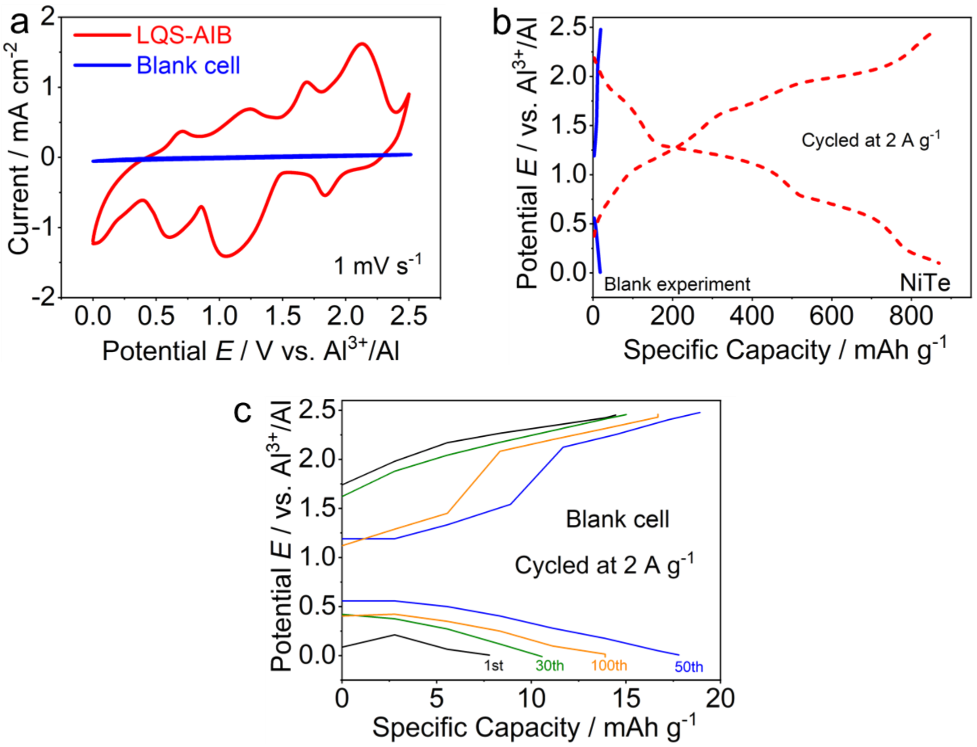

Supplementary Fig. 17. Comparison of: **a** CV and **b** charge/discharge curve between the LQS-AIB and blank cell. **c** Charge/discharge curves of the blank cell.

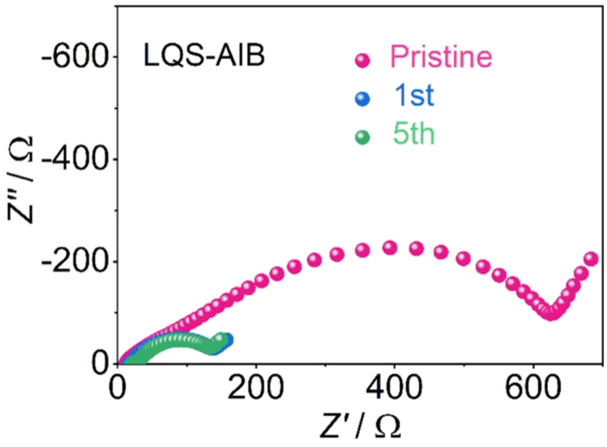

Supplementary Fig. 18. Nyquist plots of LQS-AIB after 0, 1 and 5 cycles.

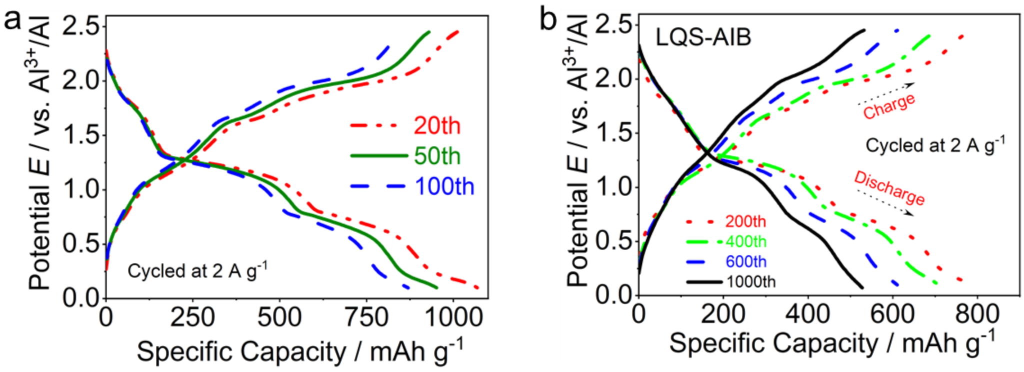

Supplementary Fig. 19. Charge and discharge curves at different cycles (20th~1000th) of LQS-AIB.

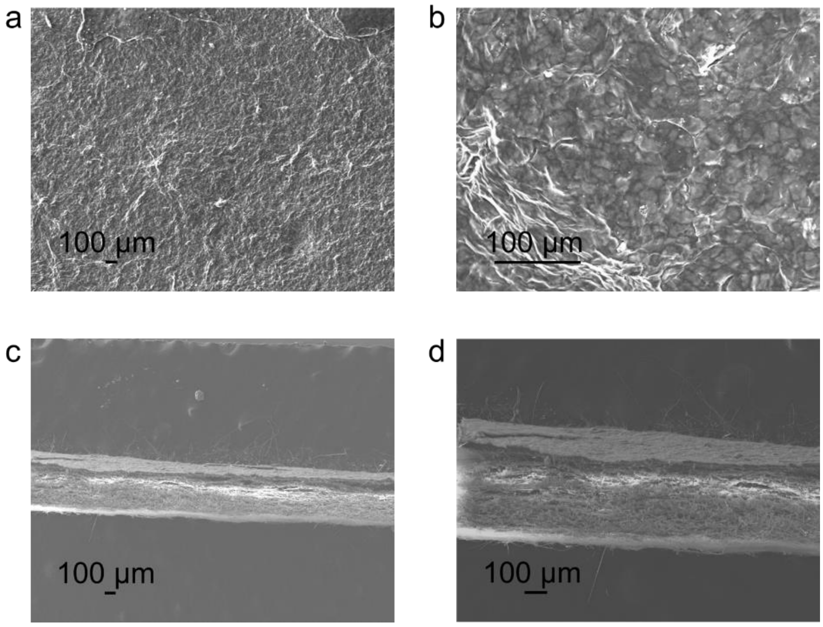

Supplementary Fig. 20. SEM images of the modified separator based on acidified SWCNTs.

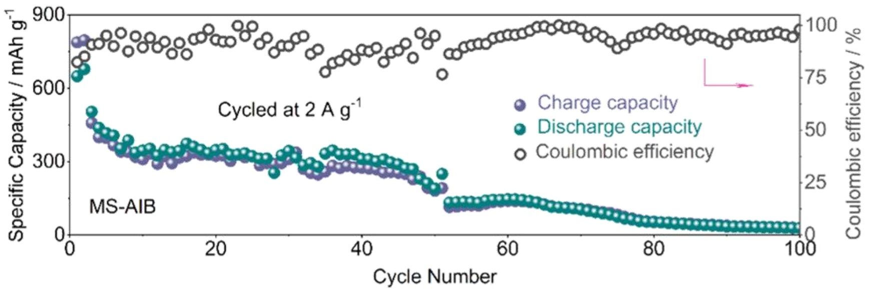

Supplementary Fig. 21. Cycling performance of AIB with modified separator (MS-AIB).

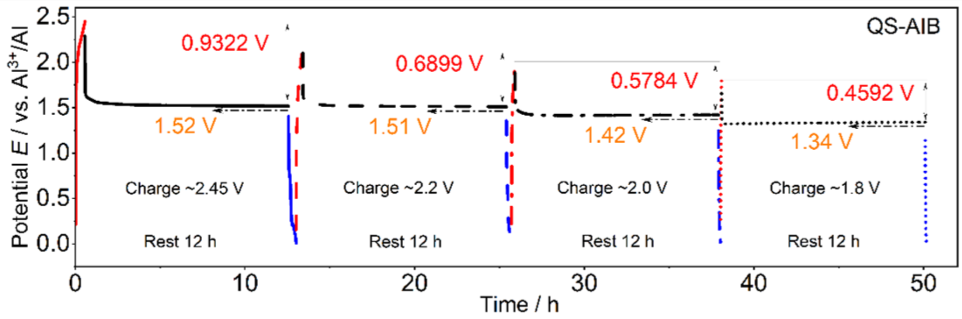

Supplementary Fig. 22. Self-discharge behaviors for different charge states of QS-AIB.

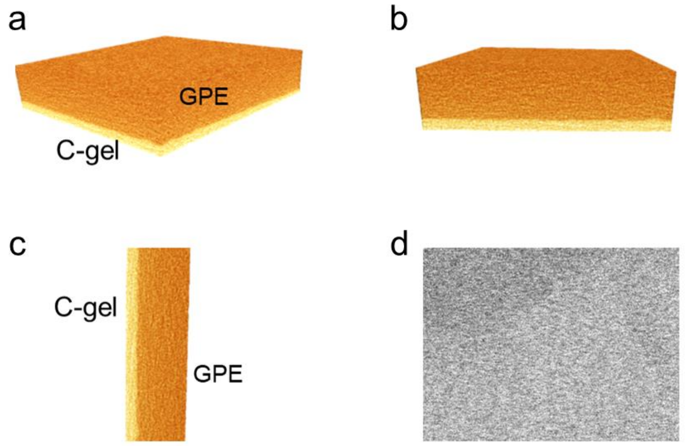

Supplementary Fig. 23. Ex-situ X-ray nano-computed tomography (CT) images of **a** reconstructed architecture of the C-gel/GPE interface in LQS-AIB and **b** front, **c** left, **d** top view of the corresponding profiles.

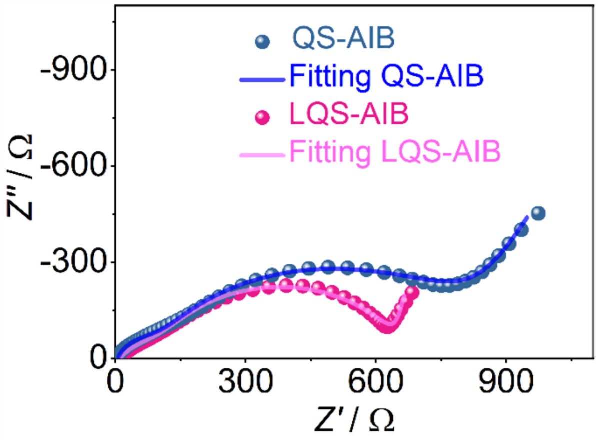

Supplementary Fig. 24. Nyquist plots of the original QS-, LQS-AIBs and the corresponding fitting curves.

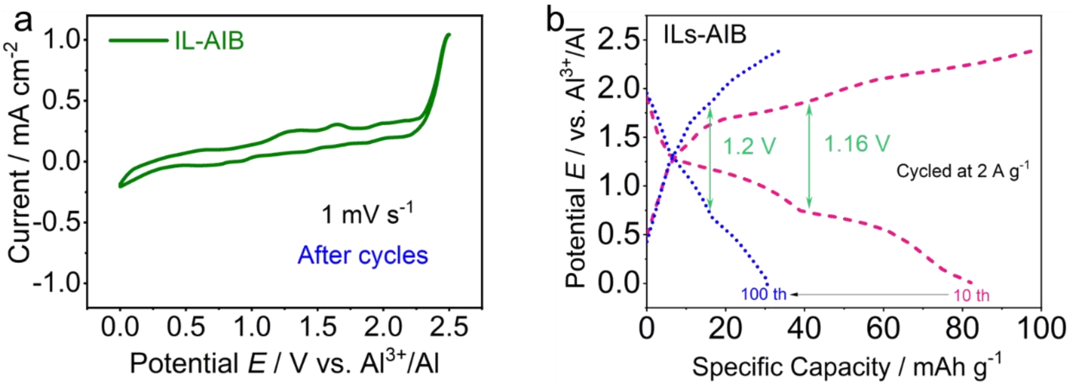

Supplementary Fig. 25. Electrochemical performance of ILs-AIB after cycling.

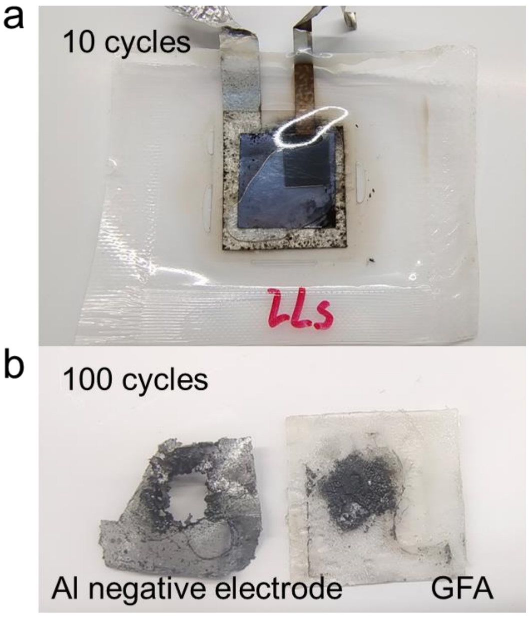

Supplementary Fig. 26. Photograph of ILs-AIB, the corresponding Al negative electrode and GF/A separator after 10 and 100 cycles.

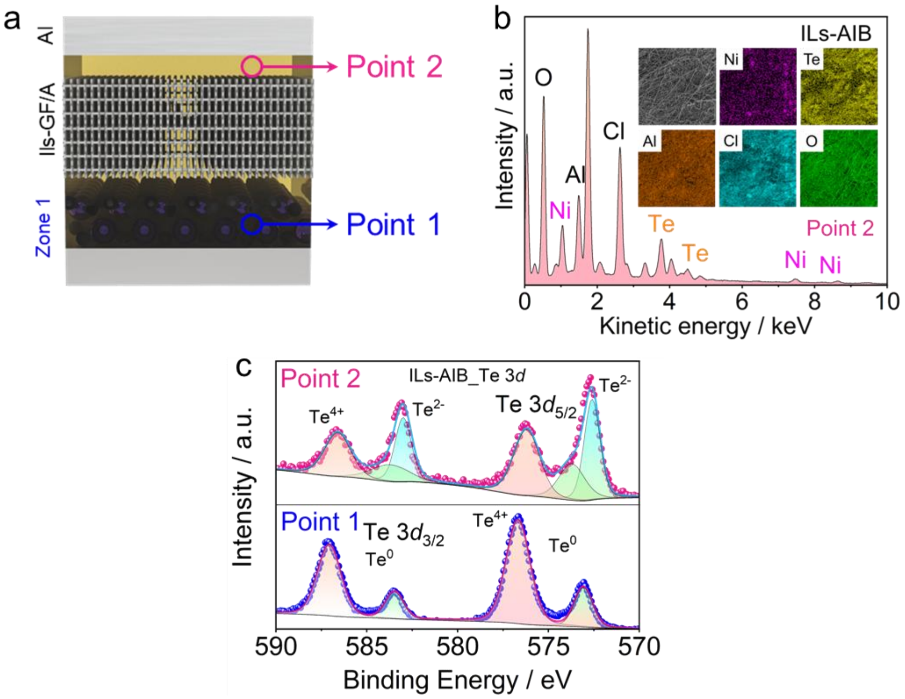

Supplementary Fig. 27. Element analysis in the cycled ILs-AIB. **a** Schematic diagram of the different points and **b** corresponding EDS spectra at point 2 and **c** XPS spectra of Te 3*d*.

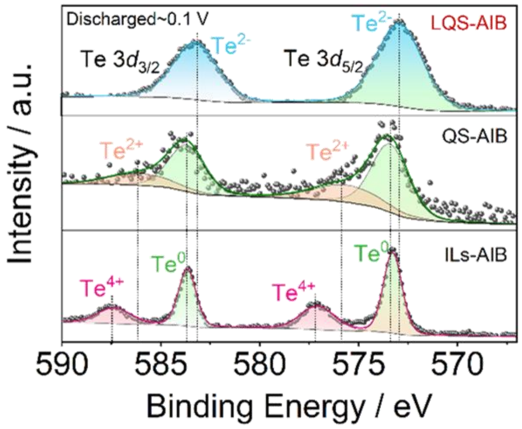

Supplementary Fig. 28. XPS spectra of Te 3*d* of the positive electrode in ILs-, QS- and LQS-AIBs at fully discharged states.

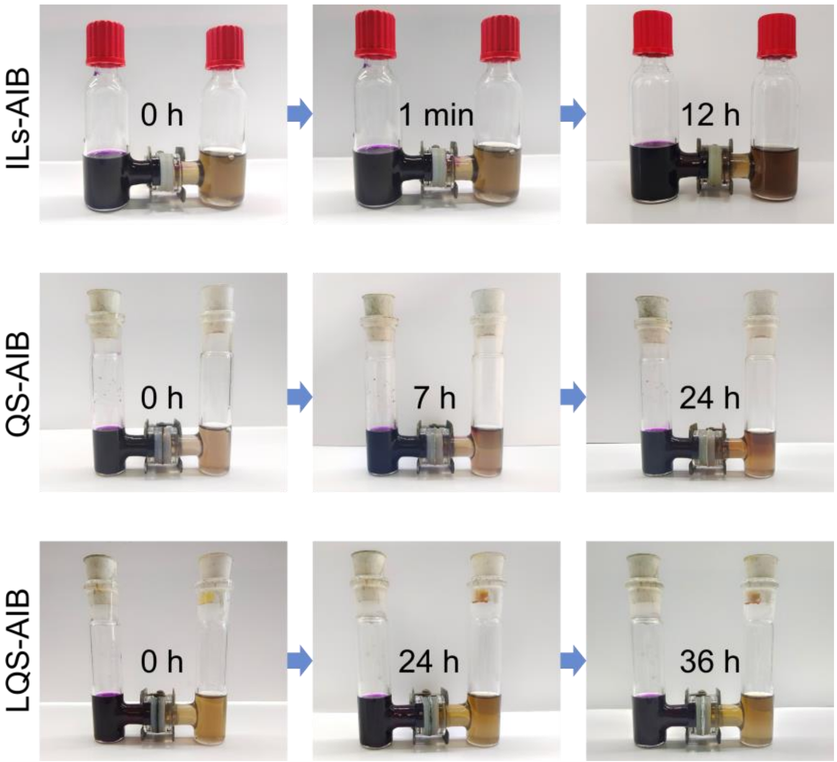

Supplementary Fig. 29. Permeation tests for the three types of AIBs.

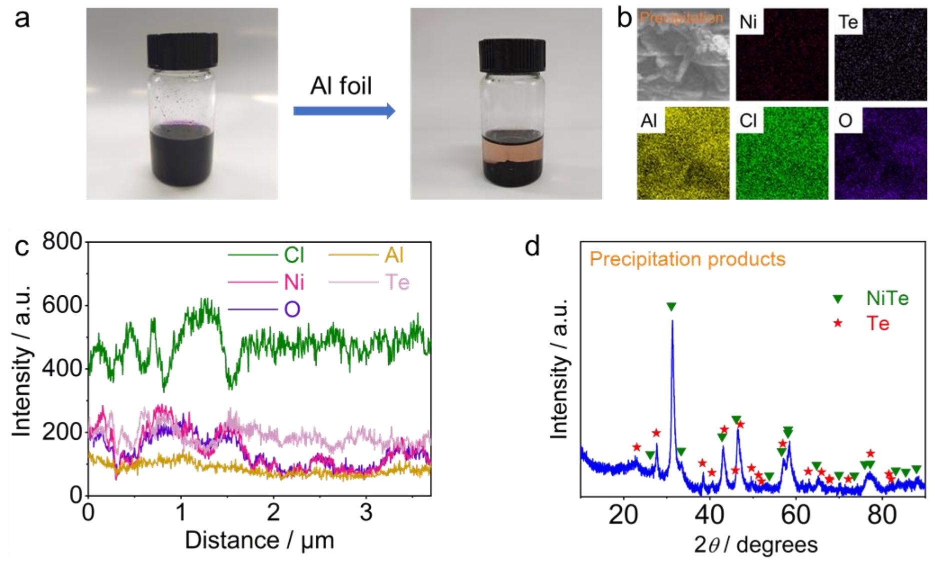

Supplementary Fig. 30. Precipitation of dissolved active materials on Al negative electrode. **a** Al foil put into the ionic liquid containing dissolved active materials. **b,c** EDS and **d** XRD spectra of the black precipitates.

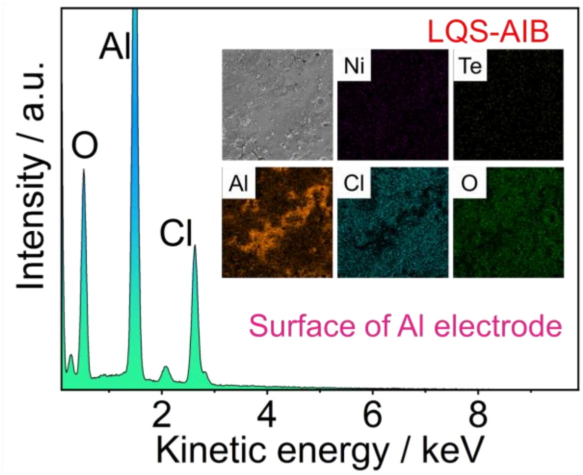

Supplementary Fig. 31. EDS spectra of Al negative electrode in LQS-AIB after cycling.

Supplementary Tables

Supplementary Table 1. Calculated total energies of the individual models and the corresponding adsorption models.

	Total energy / eV		Total energy / eV
Ni^{2+}	-41014.4978	Te^{2-}@ CNT-COOH	-284612.8447
Te^{2-}	-179975.3776	Te_4^{2+}@ CNT	-819379.5384
Te_4^{2+}	-719887.0284	Te_4^{2+}@ CNT-OH	-821440.8092
$TeCl_3^+$	-217523.9542	Te_4^{2+}@ CNT-COOH	-824523.661
$AlCl_4^-$	-56679.6343	$TeCl_3^+$@ CNT	-317012.2897
$Al_2Cl_7^-$	-100836.0476	$TeCl_3^+$@ CNT-OH	-319074.8093
CNT	-99478.78038	$TeCl_3^+$@ CNT-COOH	-322157.2076
CNT-OH	-101539.6097	$AlCl_4^-$@ CNT	-156164.1531
CNT-COOH	-104622.1648	$AlCl_4^-$@ CNT-OH	-158225.4075
Ni^{2+}@ CNT	-140513.1312	$AlCl_4^-$@ CNT-COOH	-161308.5305
Ni^{2+}@ CNT-OH	-142575.7824	$Al_2Cl_7^-$@ CNT	-200320.8184
Ni^{2+}@ CNT-COOH	-145657.9384	$Al_2Cl_7^-$@ CNT-OH	-202381.7348
Te^{2-}@ CNT	-279466.8985	$Al_2Cl_7^-$@ CNT-COOH	-205464.5305
Te^{2-}@ CNT-OH	-281529.6648		

Supplementary Table 2. Calculated binding energies of the soluble ions and the major anions in electrolyte when adsorbing on SWCNT, SWCNT-OH or SWCNT-COOH.

	Ni^{2+}	Te^{2-}	Te_4^{2+}	$TeCl_3^+$	$AlCl_4^-$	$Al_2Cl_7^-$
E_b on CNT / eV	-19.8530	-12.7405	-13.7297	-9.5551	-5.7384	-5.9904
E_b on CNT-OH / eV	-21.6749	-14.6776	-14.1712	-11.2454	-6.1635	-6.0775
E_b on CNT-COOH / eV	-21.2758	-15.3024	-14.4678	-11.0886	-6.7314	-6.3181

Supplementary Table 3. Curve fitting results of C 1*s* XPS spectra.

SWCNTs	sp^2 / %	sp^3/ %	-C-OH/ %	-COOH/ %	π-π*/ %
Original	50.92	29.85	3.97	6.06	9.20
Acidified	41.10	32.28	11.75	9.20	5.67

Supplementary Table 4. Parameters obtained by fitting the impedance spectra of the original QS- and LQS-AIBs.

	QS-AIB	LQS-AIB
R_s / Ω	5.54	11.3
R_{ct1} / Ω	84.19	115.9
CPE_1-T / F	2.44E-5	1.72E-5
CPE_1-P / F	0.86	0.67
R_{ct2} / Ω	724.3	519.2
CPE_2-T / F	1.27E-4	2.31E-4
CPE_2-P / F	0.77	0.86
Chi-squared values	0.00097	0.00076

Supplementary Table 5. Comparison of the performance of our LQS-AIB with the other Al batteries based on different positive electrode materials with various optimization strategies.

Positive electrode material	Strategy	Current density / A g^{-1}	Average voltage / V	Cycle number	Remaining capacity / mAh g^{-1}	Refs.
**NiTe**	**Double reaction regions**	2	1.3	**4000**	**>400**	**This work**
NiTe	Modified separator	0.5	1.3	100	307	[10]
Te	Heteroatom doping (N-PC-rGO)	0.5	1.5	150	467	[11]
Te	Modified TB/Al electrode	1	1.4	500	414	[12]
$NiSe_2$	3D $NiSe_2$ sponges/GO	1	1.5	250	164	[13]
Se	Se/CMK-3 in EMIBr/$AlCl_3$ ILs	0.0675	0.9	100	607	[14]
NiS	Morphology control	0.2	1.15	100	100	[15]
S	S/Co-C Electrocatalysis	1	0.6	200	500	[16]
C	Natural graphite (NG) flake film	0.66	2.0	6000	60	[17]
Cu-MOF	2D Cu-MOF/rGO	0.2	1.8	1000	155	[18]
MOF-derived carbon	Gradient N and P doping	5	1.6	2500	98	[19]
Phenanthrenequinone (PQ)	PQ triangle blending with graphite flakes	0.2	1.7	500	114	[7]

Supplementary References

1. Lin, M.-C. *et al.* An ultrafast rechargeable aluminium-ion battery. *Nature* **520**, 324-328 (2015).

2. Kravchyk, K. V. & Kovalenko, M. V. Rechargeable dual-ion batteries with graphite as a cathode: Key challenges and opportunities. *Adv. Energy Mater.* **9**, 1901749 (2019).

3. Huang, Z. *et al.* Manipulating anion intercalation enables a high-voltage aqueous dual ion battery. *Nat. Commun.* **12**, 3106 (2021).

4. Placke, T. *et al.* Perspective on performance, cost, and technical challenges for practical dual-ion batteries. *Joule* **2**, 2528-2550 (2018).

5. Zhou, X. *et al.* Strategies towards Low-Cost Dual-Ion Batteries with High Performance. *Angew. Chem. Int. Ed.* **59**, 3802-3832 (2020).

6. Kravchyk, K. V., Wang, S., Piveteau, L. & Kovalenko, M. V. Efficient aluminum chloride-natural graphite battery. *Chem. Mater.* **29**, 4484–4492 (2017).

7. Kim, D. J. *et al.* Rechargeable aluminum organic batteries. *Nat. Energy* **4**, 51–59 (2019).

8. Kravchyk, K. V. & Kovalenko, M. V. Aluminum electrolytes for Al dual-ion batteries. *Commun. Chem.* **3**, 1-9 (2020).

9. Han, X. *et al.* Electrolytes for rechargeable aluminum batteries. *Prog. Mater. Sci.* **128**, 100960 (2022).

10. Yu, Z.*, et al.* Rechargeable nickel telluride/aluminum batteries with high capacity and enhanced cycling performance. *ACS Nano* **14**, 3469-3476 (2020).

11. Zhang, X., Wang, M., Tu, J. & Jiao, S. Hierarchical N-doped porous carbon hosts for stabilizing tellurium in promoting Al-Te batteries. *J. Energy Chem.* **57**, 378-385 (2021).

12. Zhang, X. & Jiao, S. Modified Al negative electrode for stable high-capacity Al-Te batteries. *Int. J. Miner., Metall. Mater.* **29**, 896-904 (2022).

13. Zhang, H., *et al.* Evidence for dual anions co-insertion in a transition metal chalcogenide cathode material $NiSe_2$ for high-performance rechargeable aluminum-ion batteries. *Energy Storage Mater.* **47**, 336-344 (2022).

14. Liu, S., *et al.* An advanced high energy-efficiency rechargeable aluminum-selenium battery. *Nano Energy* **66**, 104159 (2019).

15. Yu, Z., Kang, Z., Hu, Z., Lu, J., Zhou, Z. & Jiao, S. Hexagonal NiS nanobelts as advanced cathode materials for rechargeable Al-ion batteries. *Chem. Commun.* **52**, 10427-10430 (2016).

16. Guo, Y., *et al.* Rechargeable aluminum-sulfur battery with improved electrochemical performance by cobalt-containing electrocatalyst. *Angew. Chem. Int. Ed.* **59**, 22963-22967 (2020).

17. Wang, D.-Y., *et al.* Advanced rechargeable aluminum ion battery with a high-quality natural graphite cathode. *Nat. Commun.* **8**, 14283 (2017).

18. Guo, Y., Wang, W., Lei, H., Wang, M. & Jiao, S. Alternate storage of opposite charges in multisites for high-energy-density Al-MOF batteries. *Adv. Mater.* **34**, 2110109 (2022).

19. Li, C., *et al.* Heteroatomic interface engineering in MOF-derived carbon heterostructures with built-in electric-field effects for high performance Al-ion batteries. *Energy Environ. Sci.* **11**, 3201-3211 (2018).
